# Supplementary material for: Exploring organic chemical space for materials discovery using crystal structure prediction-informed evolutionary optimisation
Source: Nat Commun. 2025 Nov 26;16:10540. doi: 10.1038/s41467-025-65564-8 (PMC12658106; doi:10.1038/s41467-025-65564-8)
Supplement: Supplementary file 1 — Supplementary Information [file 41467_2025_65564_MOESM1_ESM.pdf]

Supplementary Information:

Contents

Supplementary Note 1: Crystal structure prediction sub-sampling evaluations 1
Supplementary Note 2: Crystal structure mobility evaluations.. 5
Supplementary Note 3: Landscape averaged fitness evaluation 8
Supplementary Note 4: CSP methodology changes from previous study (Reorg-EA)...................... 9
Supplementary Note 5: Results from CSP informed EAs... 11
Supplementary Note 6: Marcus Theory low energy energy-structure-function maps 18
Supplementary References.. 34

Supplementary Note 1: Crystal structure prediction sub-sampling evaluations

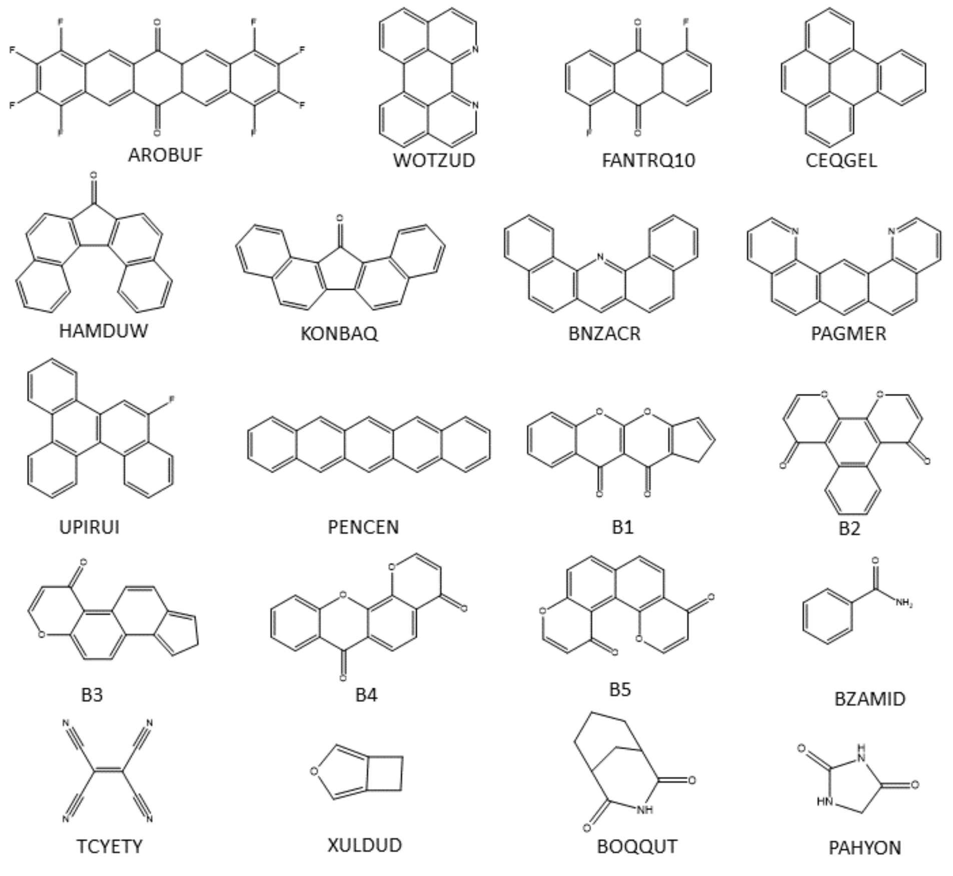

Supplementary Figure 1 - Benchmark molecules used for the evaluation of crystal structure prediction sub sampling schemes, 15 of which are experimentally synthesised molecules with known crystal structures in the CSD [1]. Of the 15, 10 are polyaromatic molecules with semiconductor-like characteristics and 5 small molecule targets. The experimental molecules are referred to by Refcode from the CSD. A further 5 molecules were then created using our evolutionary algorithm combining rules (**B1-5**).

Supplementary Table 1: Tables showing the different sampling schemes investigated with the total number of structures in each scheme and the number of times the global minimum structure from a comprehensive CSP sampling was recovered in the cut down samplings. Sampling scheme choices have been chosen based upon space group occurrences from the CSD [1], as well as the previous CSP studies in Ref. [2] and [3].

Space group	Space group Number	Cumulative % Occurrence	Sampling Schemes					
			SG14-500	SG14-1000	SG14-2000	Top5-500	Top5-1000	Top5-2000
$P2_1/c$	14	39.03	500	1000	2000	500	1000	2000
$P2_12_12_1$	19	55.99	-	-	-	500	1000	2000
$P\bar{1}$	2	72.33	-	-	-	500	1000	2000
$P2_1$	4	81.51	-	-	-	500	1000	2000
Pbca	61	86.48	-	-	-	500	1000	2000
Total Structures Sampled:			500	1000	2000	2500	5000	10000
Number of global minimum structures recovered			12	15	15	15	17	17
Percentage of low energy structures recovered (%)			25.67	29.93	33.86	43.97	48.77	52.92

Space group	Space group Number	Cumulative % Occurrence from the CSD	Sampling Schemes							
			Top10-500	Top10-1000	Top10-2000	Sampling				
						A	B	C	D	E
$P2_1/c$	14	39.03	500	1000	2000	2000	2000	1000	1000	1000
$P2_12_12_1$	19	55.99	500	1000	2000	1000	1000	1000	500	500
$P\bar{1}$	2	72.33	500	1000	2000	1000	1000	1000	500	500
$P2_1$	4	81.51	500	1000	2000	1000	1000	1000	500	500
Pbca	61	86.48	500	1000	2000	1000	1000	1000	500	500
C2/c	15	90.76	500	1000	2000	1000	500	500	500	-
$Pna2_1$	33	92.61	500	1000	2000	500	500	500	500	-
Cc	9	93.69	500	1000	2000	500	500	500	500	-
$Pca2_1$	29	94.65	500	1000	2000	500	500	500	500	-
C2	5	95.56	500	1000	2000	500	500	500	500	-
Total Structures Sampled:			5000	10000	20000	9000	8500	7500	5500	3000
Number of global minimum structures recovered			17	19	19	19	19	19	19	17
Percentage of low energy structures recovered (%)			63.79	71.11	77.06	73.57	71.76	68.27	67.45	47.90

From previous experience and testing of our CSP approach, we find that the lowest energy crystal structures on a CSP landscape are typically located early and frequently during a quasi-random search. Typically, it is the complete location of higher energy crystal structures that require more extensive sampling.[2,3] From the results in Ref. [2], which examined the convergence of the quasi-random structure generation process, it can be seen that for the trial molecules examined in the study that the number of unique structures found in the lowest 15 kJ mol^{-1} region of the landscape begins to plateau after 2000 successful minimisations. As such, this motivated the choice of 2000 structures as the largest sampling size for an individual space group (Supplementary Table 1). This choice was further supported from the benchmarking for the Top10-2000 CSP sampling which can be seen to identify 19 out of the 20 same lowest energy minima as the comprehensive search and 77.1 % of the low energy, 7.2 kJ mol^{-1}, window. The motivation behind the use of 500 structures and 1000 structures per space group then simply followed as factors of ¼ and ½ to investigate their effectiveness vs the predictable reduction in computational cost.

While the Top5-N and Top10-N subsampling schemes were based on evenly sampling each space group, we also included five subsampling schemes that also factored in the relative importance and

known differences in converging the search in different space groups. To factor in space group importance, we consider the frequencies of occurrence of each space group in the Cambridge Structural Database (CSD) [1]. However, these frequencies drop off quickly, from $P2_1/c$ at ~39 %, to the tenth most commonly occurring space group, C*2* at ~0.9 %. For example, if the total number of structures in a subsampling was 20,000, as with the Top10-2000 search, then $P2_1/c$ would sample 8,169 structures, while *C*2 would only sample 190, once the top 10 space group occurrences have been normalised. As the total number of structures was further reduced to become more affordable, such as with 9,000 total structures sampled, as with Sampling A, it would be 3,676 and 86 respectively. For certain space groups this low level of sampling is likely too low to locate the minimum structure for that specific space group, as such the minimum value of 500 is used. [3]

Therefore, to avoid very low sampling is some space groups, the Sampling-A to Sampling A to Sampling E schemes were constructed using the same sampling targets of 2000, 1000 and 500 structures per space group, but assigned based on observed frequencies in the CSD.

Supplementary Note 2: Crystal structure mobility evaluations

The evaluation of properties for the low energy predicted crystal structures of each sampled molecule require computationally inexpensive charge carrier mobility calculations. Therefore, mobility calculations were performed using Marcus theory to determine hopping rates between dimers in the crystal at 300K to generate a diffusion matrix. The Einstein relation was then applied and the eigenvalues of the resulting mobility tensor used to determine the mobility for each cartesian direction, with *Z* defined as the highest mobility direction. The most costly element of these calculations is the evaluation of the electronic coupling between molecules, for which we used the low-cost analytic overlap method (AOM) approach which has been fitted to reproduce electronic couplings between π-conjugated organic molecules. Reorganisation energies were calculated using the four-point approach.[4]

This AOM methodology is agnostic to hole or electron charge carrier transport, only requiring that molecules frontier orbitals be able to be accurately represented by combinations of its p-orbitals.[5,6] To highlight the effectiveness of this approach to rank the properties of different molecules and their adopted polymorphs, we evaluated hole mobilities for p-type organic semiconductors with known crystal structures (Supplementary Figure 2). Molecules forming p-type semiconductors were chosen with known relationships between polymorphs as they greatly outnumber n-type in literature, which is a motivating factor for targeting n-type molecules with this study, as well as to combat issues with comparison to experimentally calculated mobilities due to differences in OFET device architectures.[7,8] Additionally, rubrene and pentacene are archetypal examples of organic semiconductors, with the tetrathiafulvalene (TTF) family of molecules also being well investigated.[7]

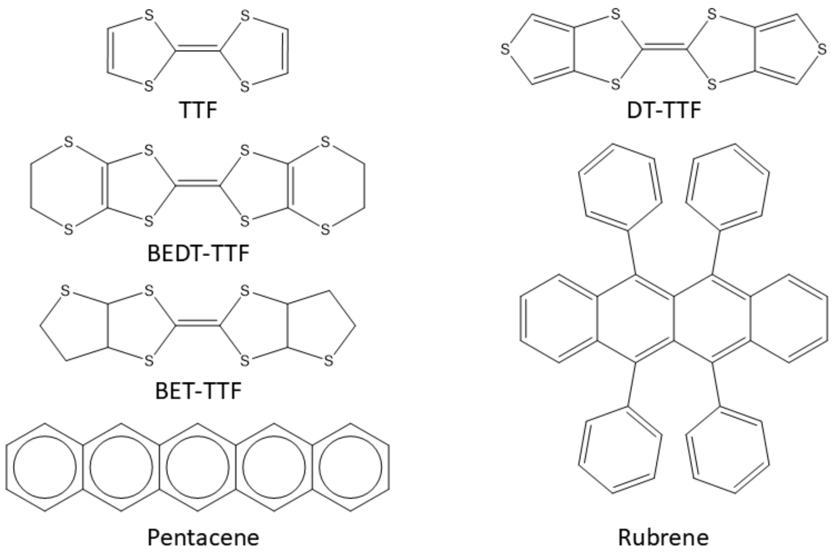

<u>Supplementary Figure 2</u> – Known p-type organic semiconducting molecules.

For each crystal the coordinates of the isolated molecule were extracted from its known crystal structure. As molecules were treated as rigid for crystal structure prediction calculations only one set of initial xyz coordinates were used for TTF, pentacene and rubrene, extracted from Refcodes BDTOLE02, PENCEN and QQQCIG01 respectively. The molecular geometries were then optimised and reorganisation energies calculated using the four-point approach with Psi4 at the B3LYP/ 6-311+G(d,p)

level of theory. The molecules in the crystal were then substituted for the now optimised geometries before the crystals were relaxed in a 3-step procedure as described in the Methods. Marcus theory charge hopping rates and overall mobilities were then calculated.

Supplementary Table 2- Table of different experimental polymorphs reported in the CSD of known p-type organic crystalline semiconductors with their average and maximum calculated mobilities using Marcus theory. Two of the known reported Pentacene polymorphs (I and II) are reported for comparison to final values from the EA search.

Crystal	Refcode	Average Mobility [$cm^2(Vs)^{-1}$]	Maximum Hopping Channel Mobility [$cm^2(Vs)^{-1}$]
DT-TTF	POKPAG	1.892	4.775
BET-TTF	POKNUY	0.995	2.215
BEDT-TTF	CIZMON03	0.006	0.011
TTF-α	BDTOLE10	2.894	6.389
TTF-β	BDTOLE02	0.552	0.819
Pentacene-I	PENCEN	2.196	4.403
Pentacene-II	PENCEN01	3.828	9.168
Rubrene-O	QQQCIG01	12.534	37.038
Rubrene-T	QQQCIG14	5.553	16.337
Rubrene-M	QQQCIG13	0.058	0.147

For the molecule TTF the α polymorph is reported experimentally as exhibiting a higher mobility than the β form [9]. It can be seen in Supplementary Table 2 this method captures that relationship between the polymorph, allowing the two crystal forms to be ranked due to the differing electronic couplings but same reorganisation energy. Indeed, the same recovery can be seen for Rubrene where experimentally it is reported that in terms of mobility the orthorhombic polymorph (Rubrene-O) > triclinic polymorph (Rubrene-T) > monoclinic polymorph (Rubrene-M).[10] DT-TTF, BET-TTF and BEDT-TTF are all crystals whose molecules are TTF derivatives. As part of an experimental study these three molecules were tested using the same OFET device architectures which reported mobility rankings as DT-TTF > BET-TTF > BEDT-TTF which again is recovered.[11] However, due to the non-conjugated regions of BET-TTF and BEDT-TTF the assumption that p-orbitals can accurately describe the HOMO can be seen to cause underprediction of some of their electronic couplings and therefore dimer hopping rates (Supplementary Figure 3).

As can be seen in Supplementary Table 2 the average mobilities reported can differ greatly from the calculated maximum mobilities along hopping channels in the molecules. Therefore, future study could focus on optimising 1D mobility materials for applications in thin film materials. However, as experimentally this would require more crystallisation control isotropic averaged values per crystal structure were used in this study in order to target generally high performing molecules.

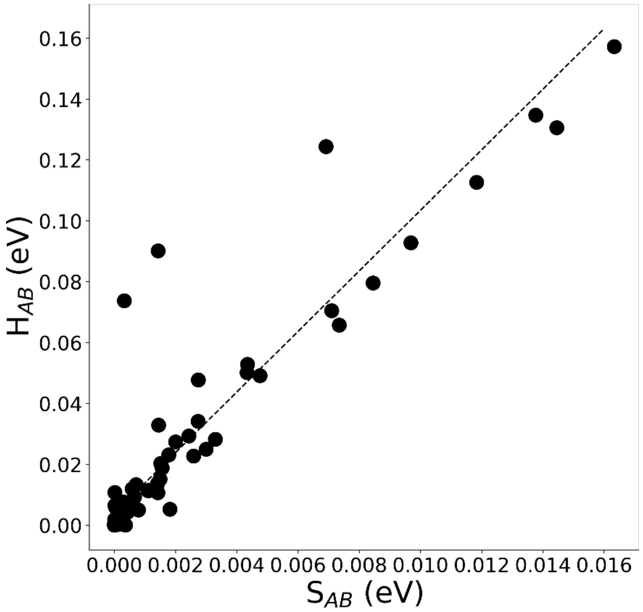

Supplementary Figure 3: Correlation between sPOD electronic couplings H_{AB} and AOM overlap integral (S_{ab}) using 10 randomly chosen unique dimers from the optimised molecule replaced crystal structures. The largest data points with the largest deviation from the line of best fit are dimers consisting of the BET-TTF and BEDT-TTF molecules.

Supplementary Note 3: Landscape averaged fitness evaluation

As a weighted average, whilst normalising the fact that different molecules have varying numbers of possible crystal structures in the low energy region for comparisons between molecules, the fitness for a specific molecule can be skewed due to the increasing number of structures at increasingly higher energies from the global minimum. This is simply due to the fact that most crystal structures predicted will not be experimentally accessible and therefore will be higher in energy. This relationship can be seen for the 7.2 kJ mol^{-1} low energy comprehensive CSP landscapes for all the molecules evaluated (Supplementary Note 6).

To counter act this bias towards the higher energy and therefore less likely to be stable structures, a calculation of the probability density was introduced for each structure using a gaussian kernel for kernel density estimation (KDE) included as $f(E_i)$. The bandwidth for the kernel, which is a smoothing parameter, is calculated for each landscape using leave one out cross validation within a calculated range of 0.05 to 1 kJ mol^{-1} to alleviate the chance of over or underfitting the data.

The influence of KDE was evaluated by converting each structure within a 7.2 kJ mol^{-1} window of the global minimum of the benchmark comprehensive CSPs into a probability of selection using the fitness function both with and without the $f(E_i)$ term, not including property values (e.g. $p_i = 1$).

The calculated probabilities were then used to randomly select 1000 structures, allowing repeats from each CSP, mimicking the chance of a crystal being synthesised and saving the energies relative to their respective global minimum. This was done in line with the polymorph paper from which the exponential function was fitted.[12] As can be seen, the exponential decay relationship is recovered with the $f(E_i)$ version of the equation unlike in the original version and therefore this form of the fitness function has been adopted for all following calculations.

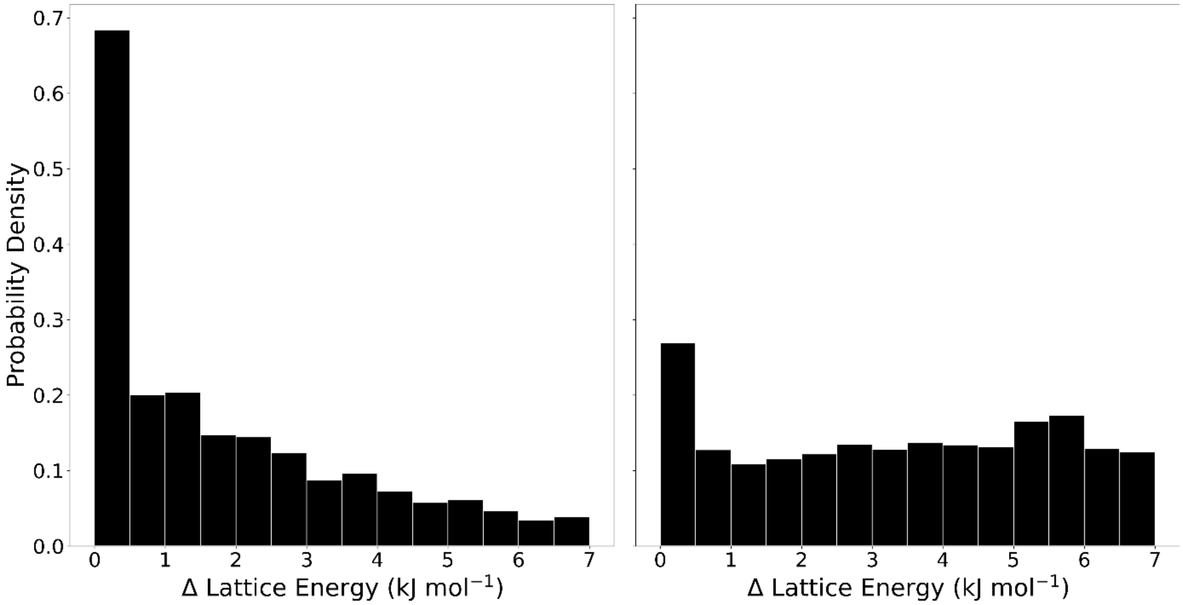

Supplementary Figure 4 – Combined histograms of the relative energies of each randomly selected crystal structure to their global minimum for both the $f(E_i)$ included (left) probability calculation method and not included (right).

Supplementary Note 4: CSP methodology changes from previous study (Reorg-EA)

For the top 10 molecules in the previous study CSP was performed using a sub sampling scheme targeting 4000 energy-minimised Z' = 1 structures in $P2_1/c$, $P\bar{1}$, $C2/c$, $P2_12_12_1$, $P2_1$, Pbca and 2000 in P1, C2, Cc, $Pna2_1$ and $Pca2_1$. This sampling scheme however is too costly for inclusion in the EA procedure and used the W99 intermolecular atom-atom potential in place of the FIT empirically parametrized exp-6 repulsion-dispersion model used in this study. As part of previous benchmarking efforts, it was found that FIT outperformed W99 and so was chosen for this study. [13] Additionally, for portability across high performance computing resources Psi4 was used for this work opposed to Gaussian09 [14] used previously. As the energetic models are different so too are expected sampled crystal structures and rankings. This subjectivity to the CSP model used further highlights the benefits of the landscape averaging EA, opposed to only considering the global minimum structure. A comparison of calculated reorganisation values using the four-point approach can be seen below.

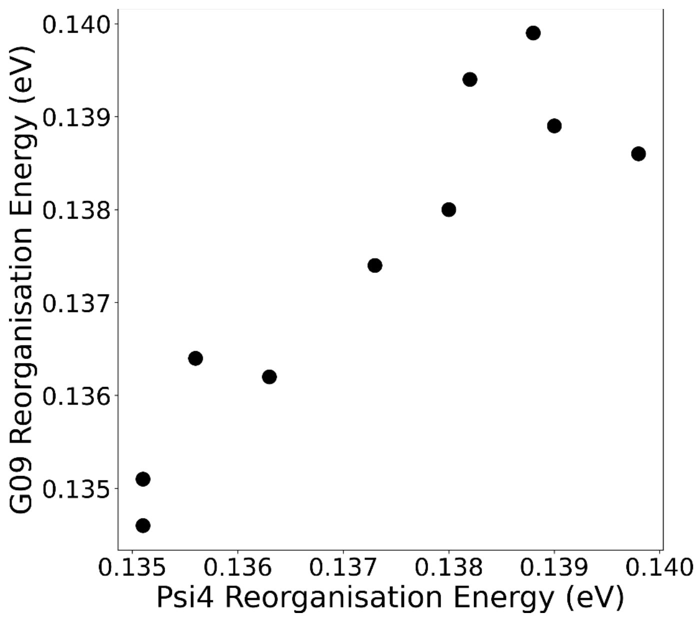

Supplementary Figure 5 – Comparison of reorganisation energies using the four-point method for the top 10 molecules from the previous study (**1A-10A**) calculated using Psi4 and Gaussian09

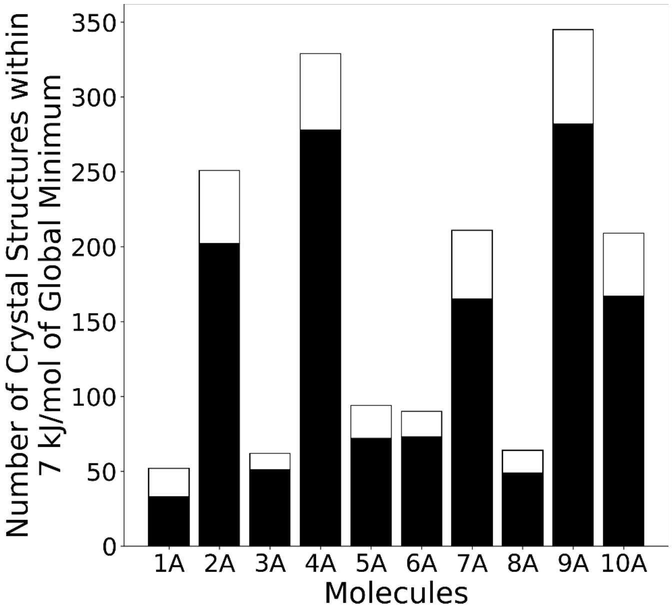

Supplementary Figure 6 – Comparison of the aggregated number of crystal structures within 7 kJ mol^{-1} of the global minimum found for **1A-10A** for the previous studies W99 landscape and the FIT re-evaluated comprehensive CSP landscape from this study trimmed down to match the sampling performed in the previous study. Trimming was performed based on quasi-random seed number for each space group sampled individually before duplicate removal as due to the quasi-random CSP search, using a sobol vector to sample degrees of freedom, sampling N crystal structures or trimming down to N in a space group is effectively the same. The shaded portion of each bar indicates the number of minimised crystal structures found on both landscapes when evaluated by comparing molecular clusters for each of size 30 molecules.

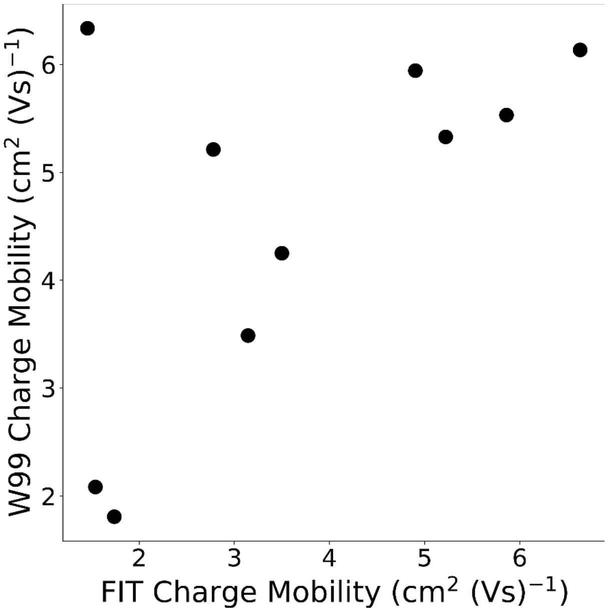

Supplementary Figure 7 – Comparison of landscape averaged mobilities in the 7 kJ mol^{-1} low energy window calculated using AOM electronic couplings with Marcus theory on the CSP sampling from the previous study for FIT and W99 CSP landscapes.

Supplementary Note 5: Results from CSP informed EAs

Whilst azapentacene molecules were included in the HAB79 dataset used to parameterise AOM, in order for greater confidence in the EA search sPOD calculations were performed on the global minimum crystal structures from the comprehensive CSP landscapes for molecules **M1-M21.** Following the same procedure as the Marcus theory hopping rate calculations all the dimers in the low energy 7.2 kJ mol^{-1} window were identified for the structure. Duplicates in the set were removed resulting in 199 unique dimers on which sPOD calculations were performed. As can be seen below the linear relationship is recovered, with a calculated *C* value of 11.206 eV from the fitting. As this value is greater than that of the universal scaling constant, 9.463 eV, it suggests the predicted couplings and mobility values are marginally underpredicted from the EA search. However, as stated the ranking is what is considered not the quantitative difference between molecules so any effect of this should be minimal.

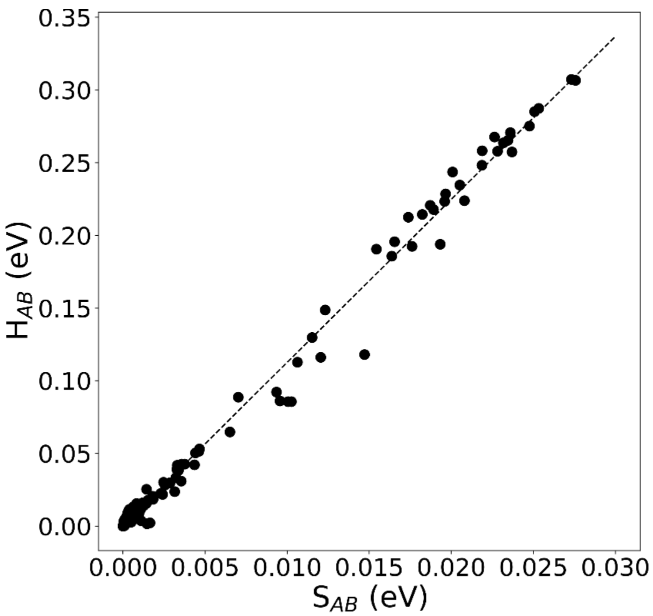

Supplementary Figure 8 – Correlation between sPOD electronic couplings H_{AB} and AOM overlap integral (S_{ab}) using the global minimum structure unique dimers for molecules **M1-21.**

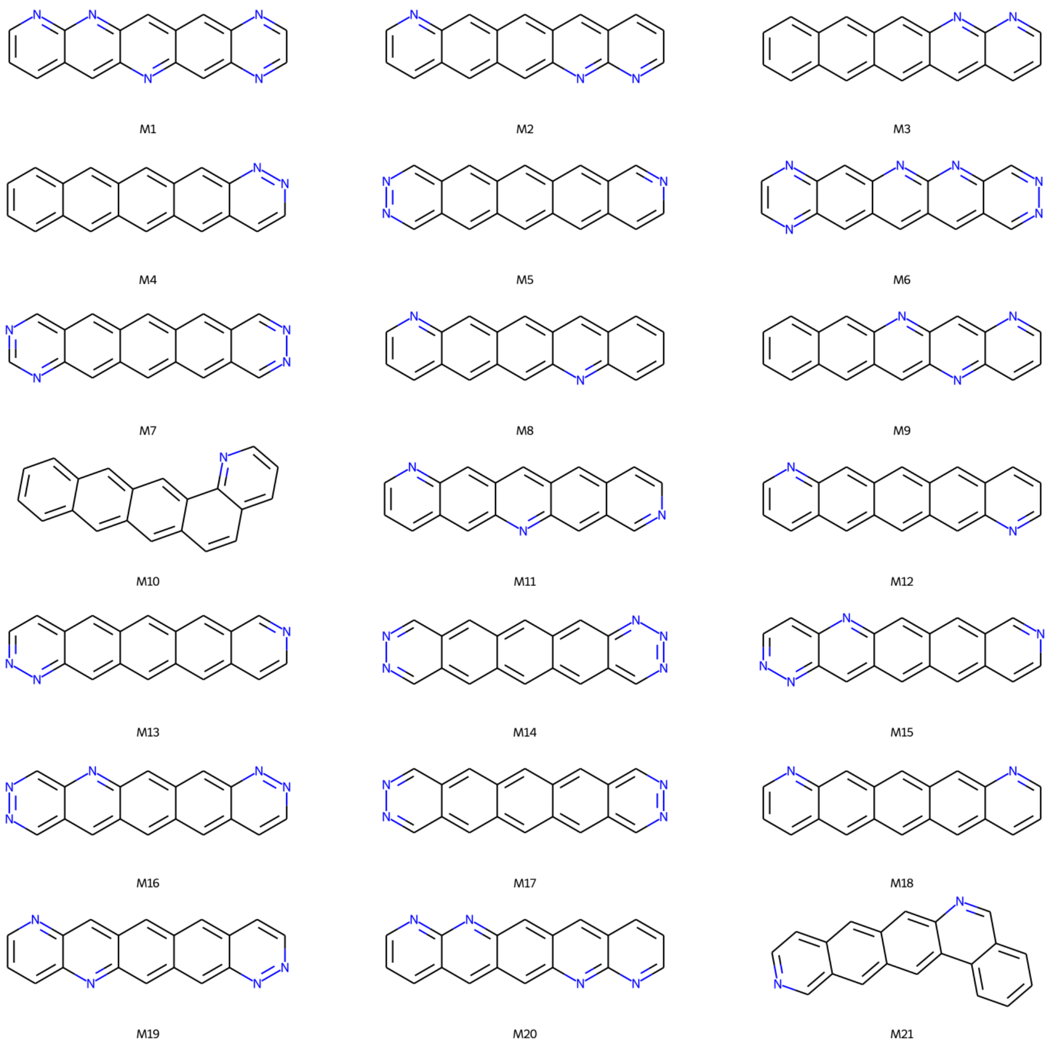

Supplementary Figure 9 – Aggregated top 10 molecules found using CSP informed EAs from targeting high mobility global minimum structures as well as for landscape averaged sub-sampling scheme evaluations.

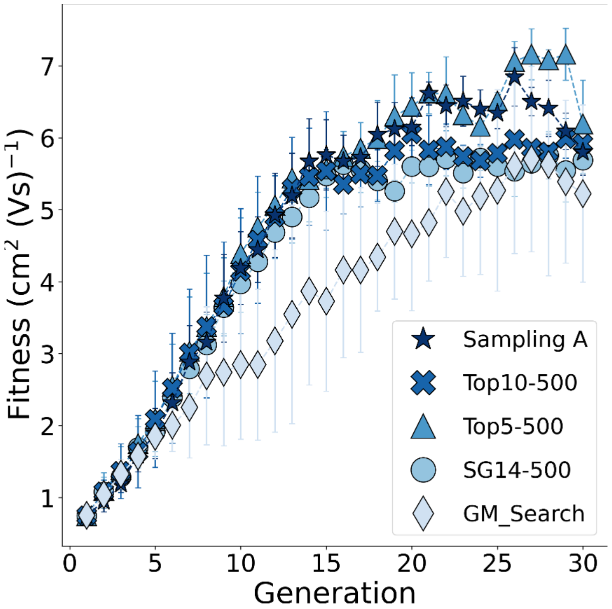

Supplementary Figure 10 – Average mobility values calculated using Marcus theory for each generation from the EA averaged across the 3 repeats for each CSP fitness evaluation method. These show that the runs were approaching or at a converged point by 30 generations. Data points show the mean over three repeats, while the bars show the variance across the three repeats. Supplementary Figures 11-15 show each repeated runs individual convergence.

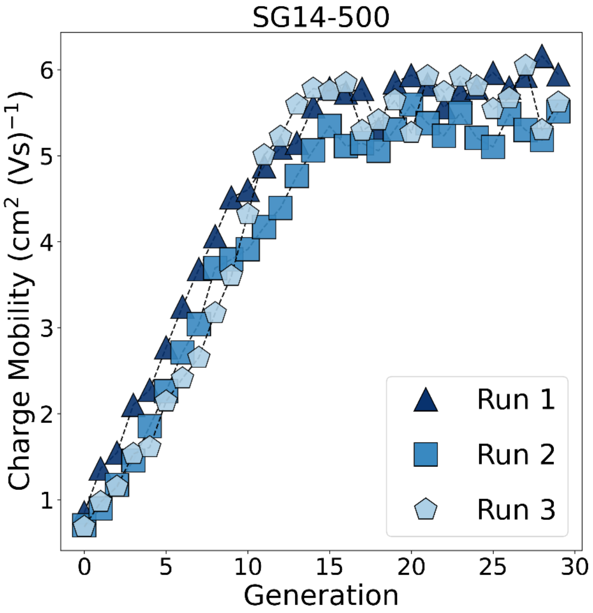

Supplementary Figure 11 – Average mobility values calculated using Marcus theory for each generation for each repeat of the SG14-500 EA.

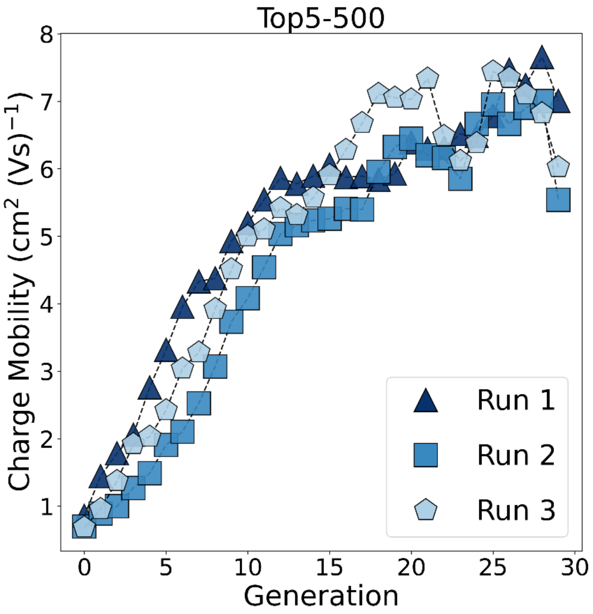

Supplementary Figure 12 – Average mobility values calculated using Marcus theory for each generation for each repeat of the Top5-500 EA.

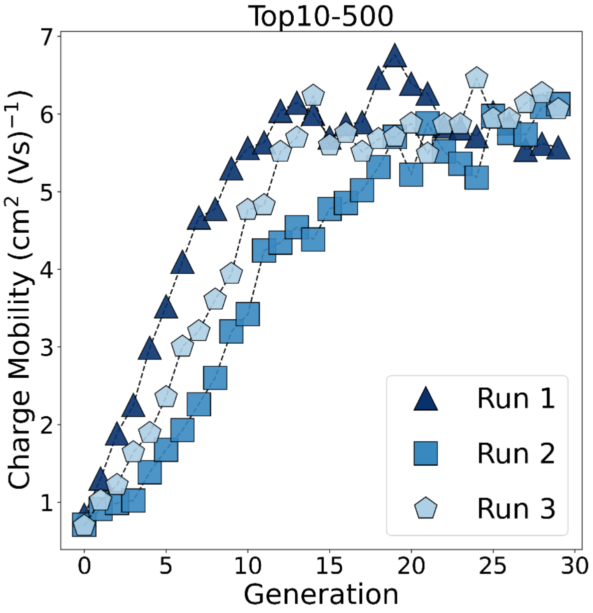

Supplementary Figure 13 – Average mobility values calculated using Marcus theory for each generation for each repeat of the Top10-500 EA.

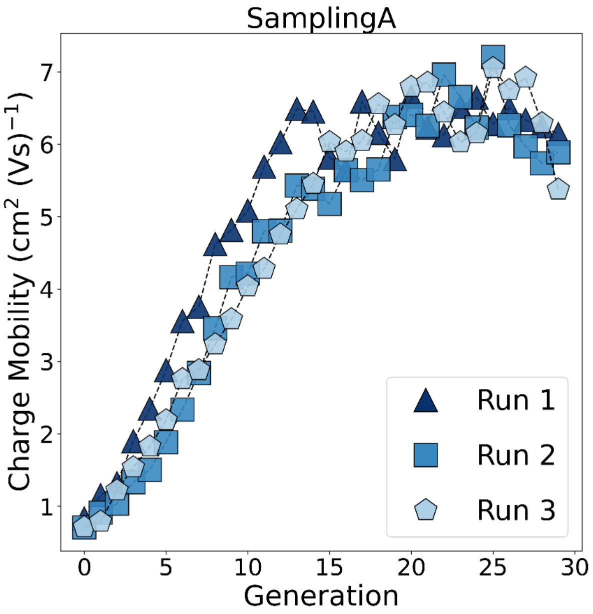

Supplementary Figure 14 – Average mobility values calculated using Marcus theory for each generation for each repeat of the Sampling A EA.

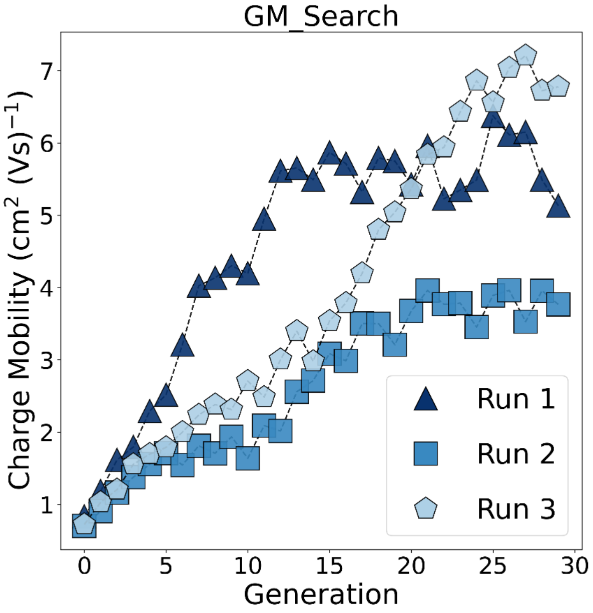

Supplementary Figure 15 – Average mobility values calculated using Marcus theory for each generation for each repeat of the global minimum targeting EA.

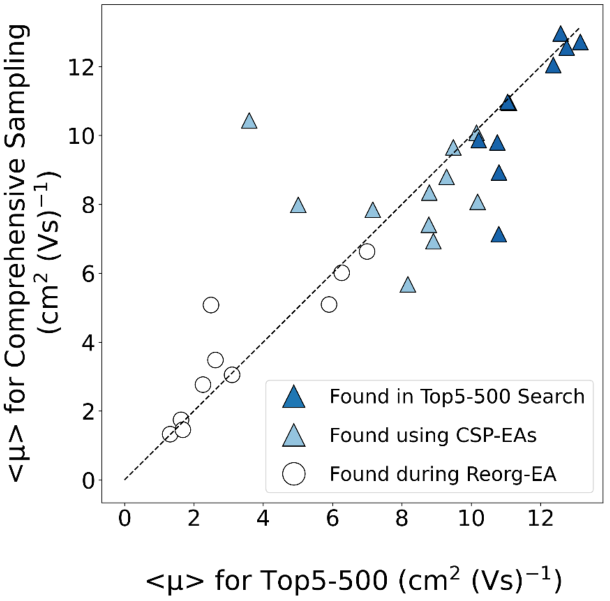

<u>Supplementary Figure 16</u> – Comprehensive CSP landscape averaged mobilities for all the top 10 molecules from each search landscape averaged mobilities at the Top5-500 sub-sampling

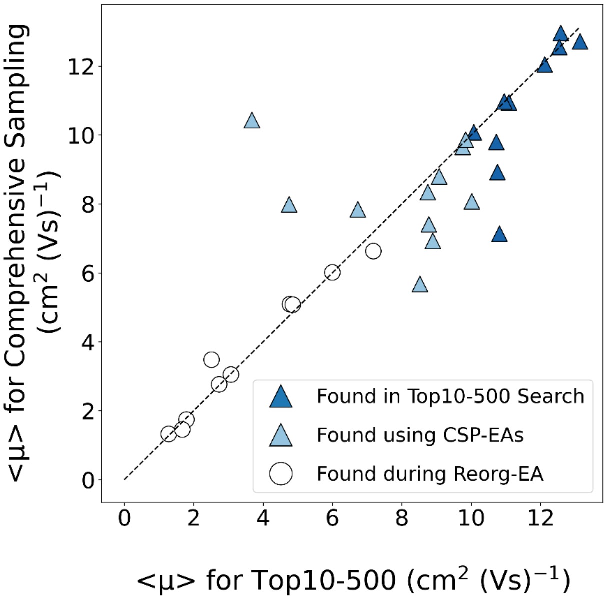

<u>Supplementary Figure 17</u> – Comprehensive CSP landscape averaged mobilities for all the top 10 molecules from each search landscape averaged mobilities at the Top5-500 sub-sampling

Supplementary Table 3 – The Root Mean Squared Error (RMSE), Mean Signed Difference (MSD), Mean Absolute Error (MAE), Kendal rank correlation (τ) and R^2 for each of the top 31 molecules whose mobility was assessed at the comprehensive CSP level against the CSP-EA CSP sampling level or reorganisation energy.

Search Setting	RMSE $[cm^2(Vs)^{-1}]$	MSD $[cm^2(Vs)^{-1}]$	MAE $[cm^2(Vs)^{-1}]$	τ	R^2
Reorg-EA	-	-	-	0.35	-
SG14-500	2.6	-0.53	1.86	0.52	0.39
Top5-500	1.8	0.12	1.09	0.75	0.71
Top10-500	1.75	0.12	0.97	0.74	0.73
Sampling A	0.6	0.2	0.3	0.91	0.97

Supplementary Table 4 – Table showing for each of the evaluated search settings the number of the aggregated top 10 molecules found for each search setting found in each run. The generation the highest fitness molecule from the 3 aggregated CSP-EA repeats was found is also shown if it was found.

Search	Run					
	1		2		3	
	Number of top 10 molecules found in the run	Generation best molecule found	Number of top 10 molecules found in the run	Generation best molecule found	Number of top 10 molecules found in the run	Generation best molecule found
SG14-500	4	-	6	14th	8	7th
Top5-500	10	15th	5	13th	9	12th
Top10-500	8	9th	4	-	7	15th
Sampling A	7	9th	8	14th	7	28th
GM_Search	8	-	2	17th	3	-

Supplementary Note 6: Marcus Theory low energy energy-structure-function maps

Marcus theory calculated energy-structure-function maps of the 7.2 kJ mol^{-1} low energy window for molecules **M1-21** AND **1A-10A** on comprehensive CSP sampling. On each landscape each data point represents a sampled crystal structure, with the darker and larger the data point the greater the calculated mobilities. Each structure is also classified by its packing motif.

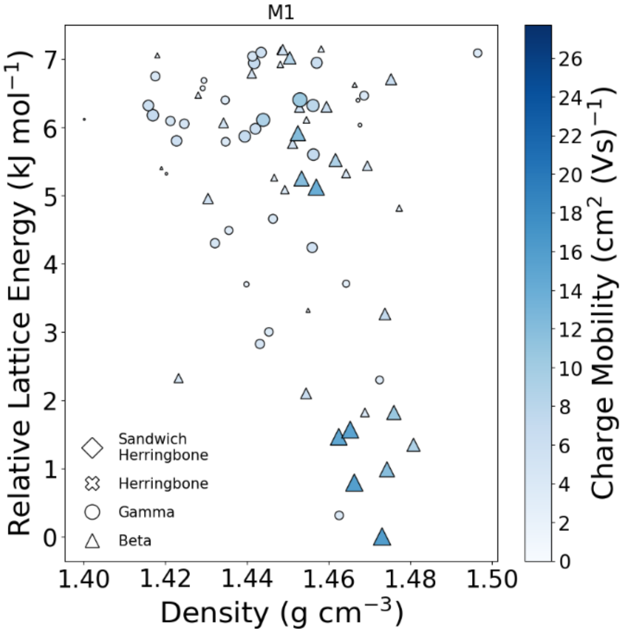

Supplementary Figure 18 – Predicted 7.2 kJ mol^{-1} low energy window energy-density-function map for charge mobilities of each crystal structure for **M1**. Each data point represents a sampled crystal structure, with the darker and larger the data point the greater the calculated mobilities. Each structure is also classified by its packing motif.

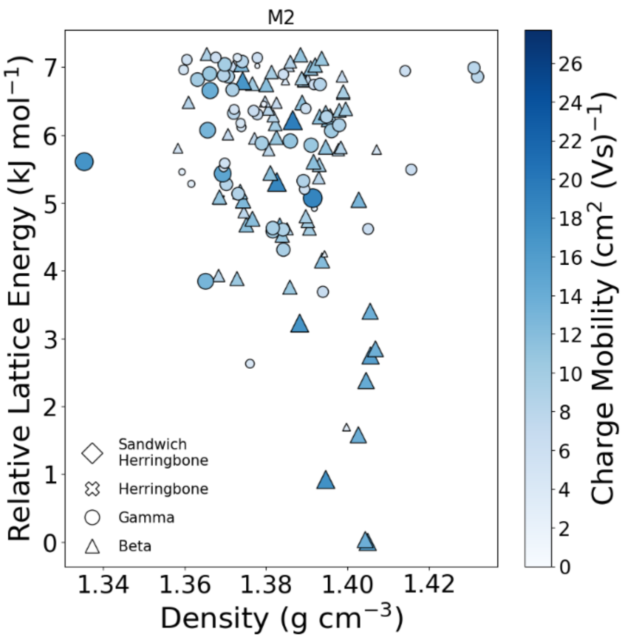

Supplementary Figure 19 – Predicted 7.2 kJ mol^{-1} low energy window energy-density-function map for charge mobilities of each crystal structure for **M2**. Each data point represents a sampled crystal structure, with the darker and larger the data point the greater the calculated mobilities. Each structure is also classified by its packing motif.

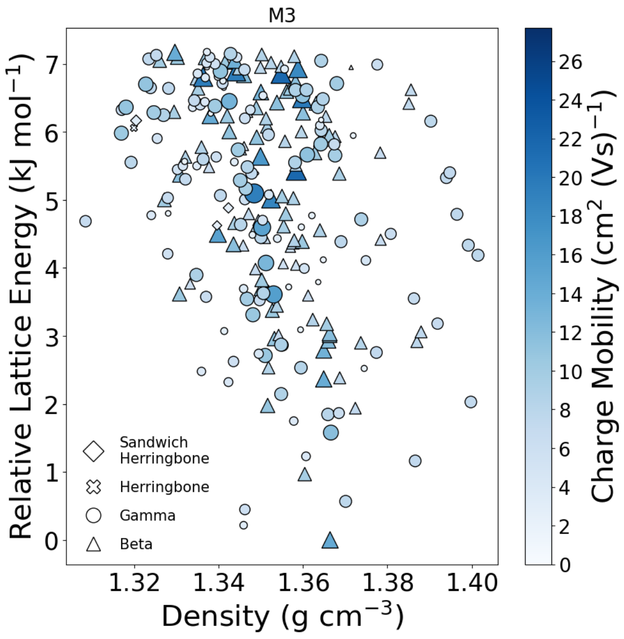

Supplementary Figure 20 – Predicted 7.2 kJ mol^{-1} low energy window energy-density-function map for charge mobilities of each crystal structure for **M3**. Each data point represents a sampled crystal structure, with the darker and larger the data point the greater the calculated mobilities. Each structure is also classified by its packing motif.

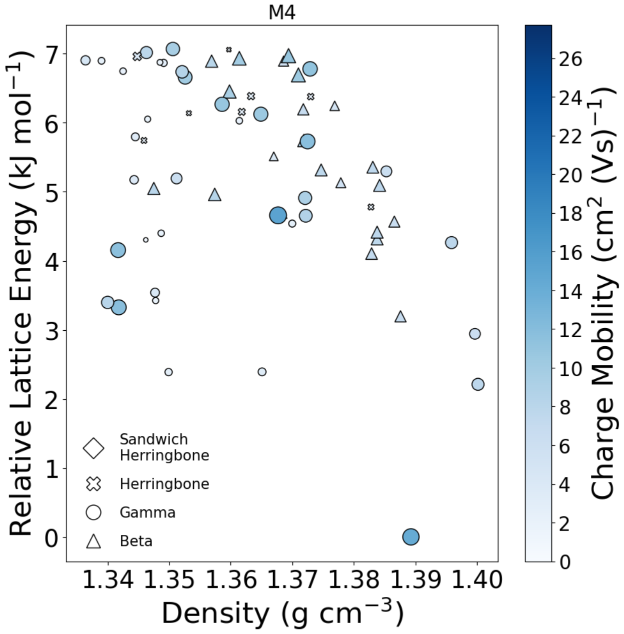

Supplementary Figure 21 – Predicted 7.2 kJ mol^{-1} low energy window energy-density-function map for charge mobilities of each crystal structure for **M4**. Each data point represents a sampled crystal structure, with the darker and larger the data point the greater the calculated mobilities. Each structure is also classified by its packing motif.

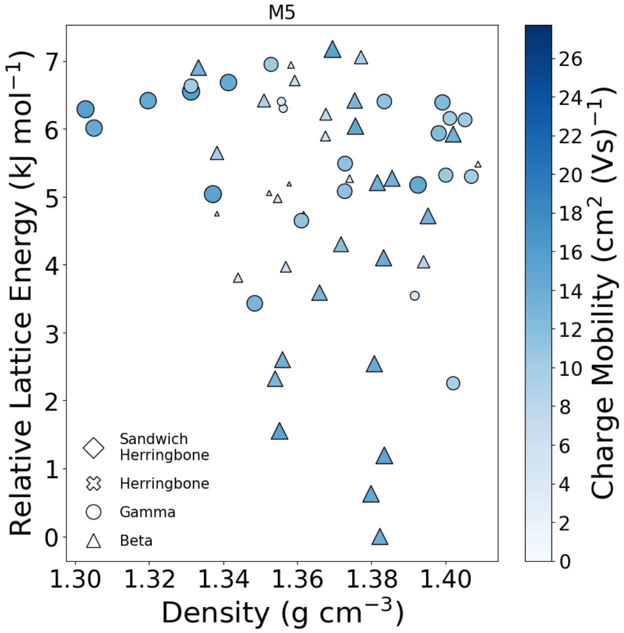

Supplementary Figure 22 – Predicted 7.2 kJ mol^{-1} low energy window energy-density-function map for charge mobilities of each crystal structure for **M5**. Each data point represents a sampled crystal structure, with the darker and larger the data point the greater the calculated mobilities. Each structure is also classified by its packing motif.

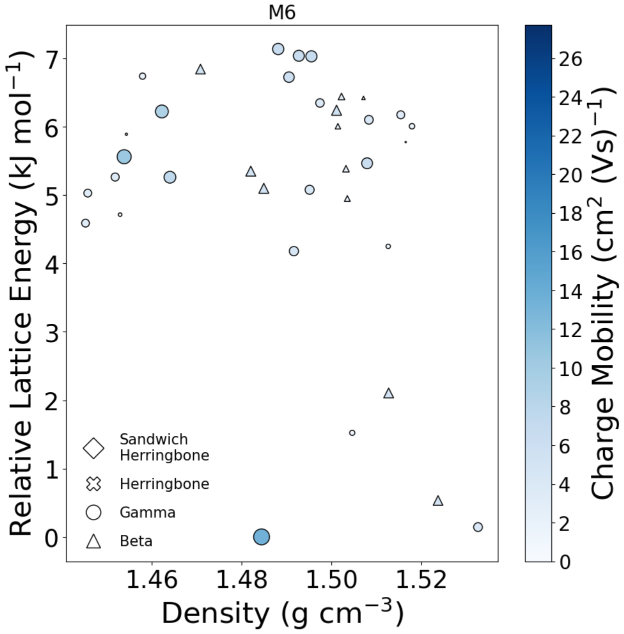

Supplementary Figure 23 – Predicted 7.2 kJ mol^{-1} low energy window energy-density-function map for charge mobilities of each crystal structure for **M6**. Each data point represents a sampled crystal structure, with the darker and larger the data point the greater the calculated mobilities. Each structure is also classified by its packing motif.

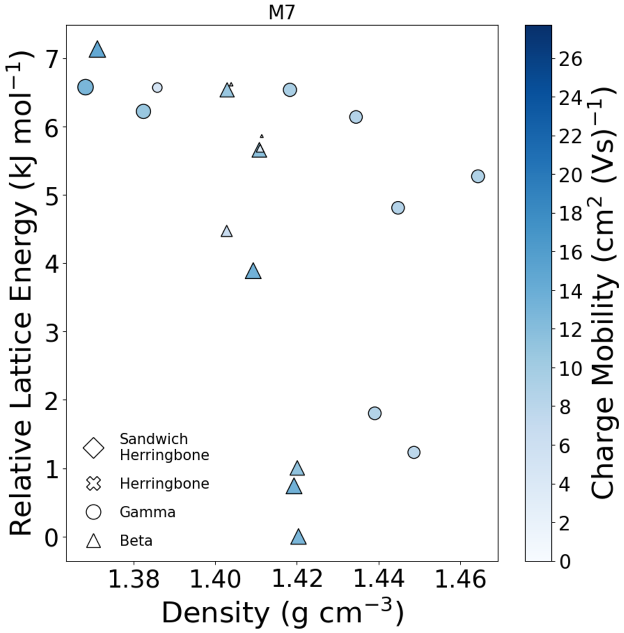

Supplementary Figure 24 – Predicted 7.2 kJ mol^{-1} low energy window energy-density-function map for charge mobilities of each crystal structure for **M7**. Each data point represents a sampled crystal structure, with the darker and larger the data point the greater the calculated mobilities. Each structure is also classified by its packing motif.

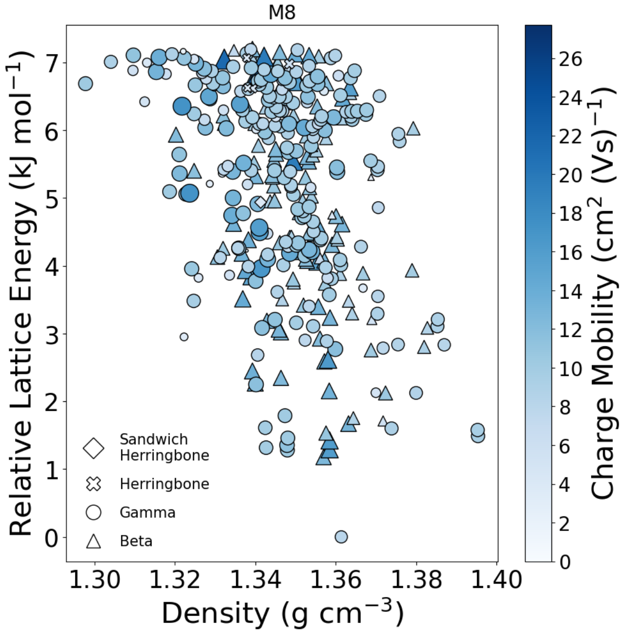

Supplementary Figure 25 – Predicted 7.2 kJ mol^{-1} low energy window energy-density-function map for charge mobilities of each crystal structure for **M8**. Each data point represents a sampled crystal structure, with the darker and larger the data point the greater the calculated mobilities. Each structure is also classified by its packing motif.

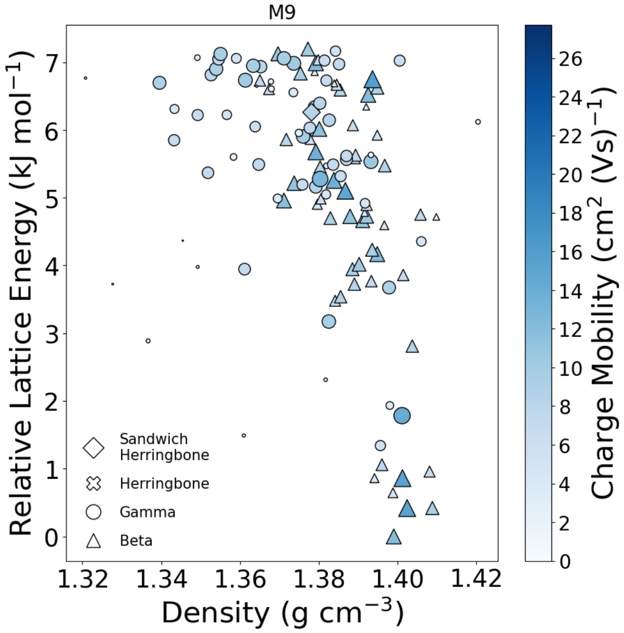

Supplementary Figure 26 – Predicted 7.2 kJ mol^{-1} low energy window energy-density-function map for charge mobilities of each crystal structure for **M9**. Each data point represents a sampled crystal structure, with the darker and larger the data point the greater the calculated mobilities. Each structure is also classified by its packing motif.

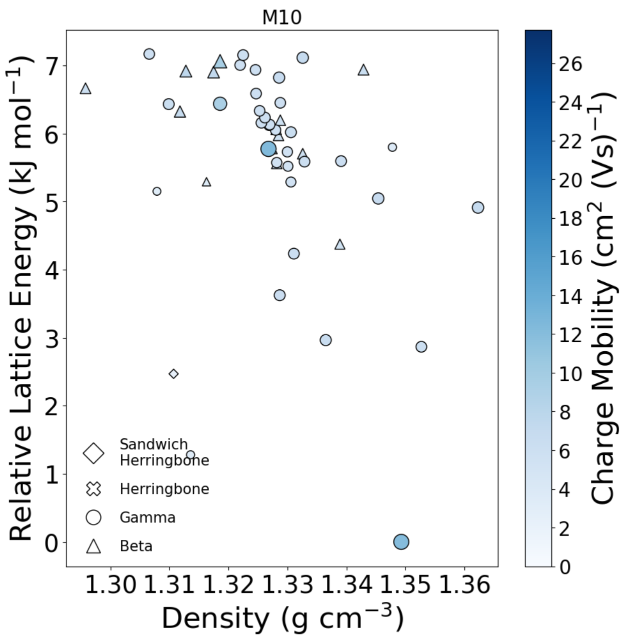

Supplementary Figure 27 – Predicted 7.2 kJ mol^{-1} low energy window energy-density-function map for charge mobilities of each crystal structure for **M10**. Each data point represents a sampled crystal structure, with the darker and larger the data point the greater the calculated mobilities. Each structure is also classified by its packing motif.

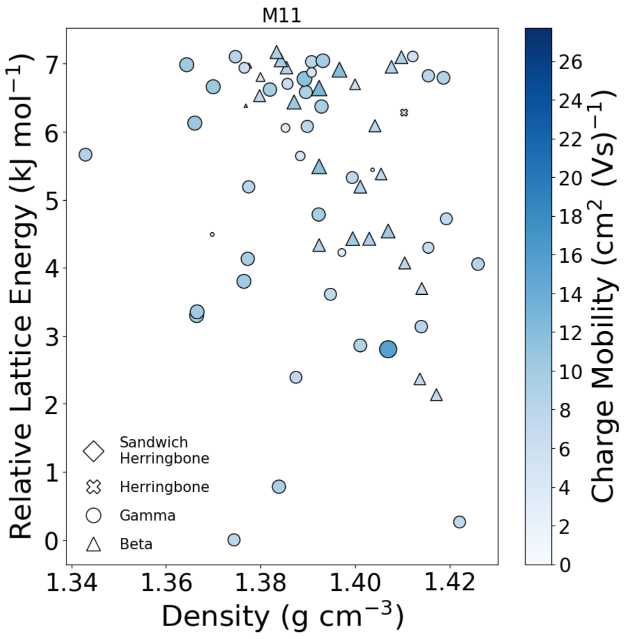

Supplementary Figure 28 – Predicted 7.2 kJ mol^{-1} low energy window energy-density-function map for charge mobilities of each crystal structure for **M11**. Each data point represents a sampled crystal structure, with the darker and larger the data point the greater the calculated mobilities. Each structure is also classified by its packing motif.

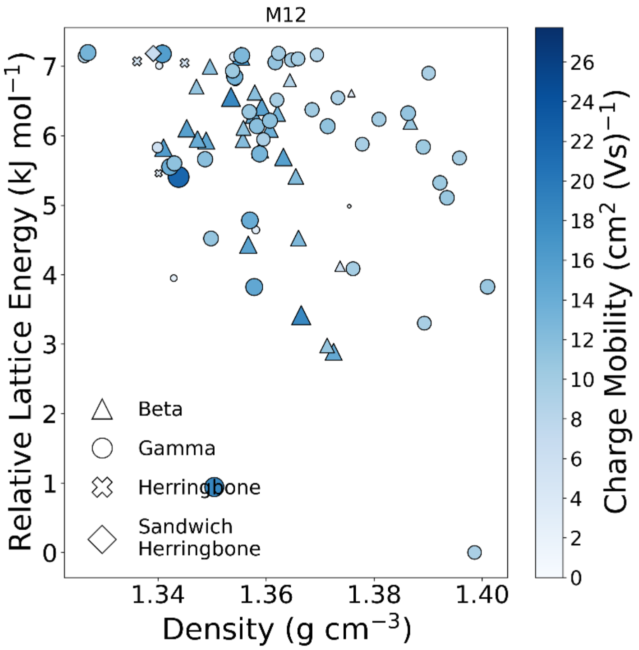

Supplementary Figure 29 – Predicted 7.2 kJ mol^{-1} low energy window energy-density-function map for charge mobilities of each crystal structure for **M12**. Each data point represents a sampled crystal structure, with the darker and larger the data point the greater the calculated mobilities. Each structure is also classified by its packing motif.

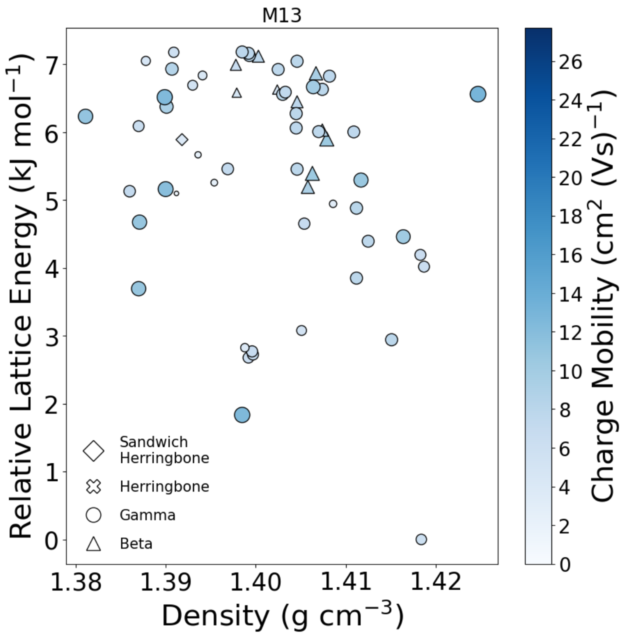

Supplementary Figure 30 – Predicted 7.2 kJ mol^{-1} low energy window energy-density-function map for charge mobilities of each crystal structure for **M13**. Each data point represents a sampled crystal structure, with the darker and larger the data point the greater the calculated mobilities. Each structure is also classified by its packing motif.

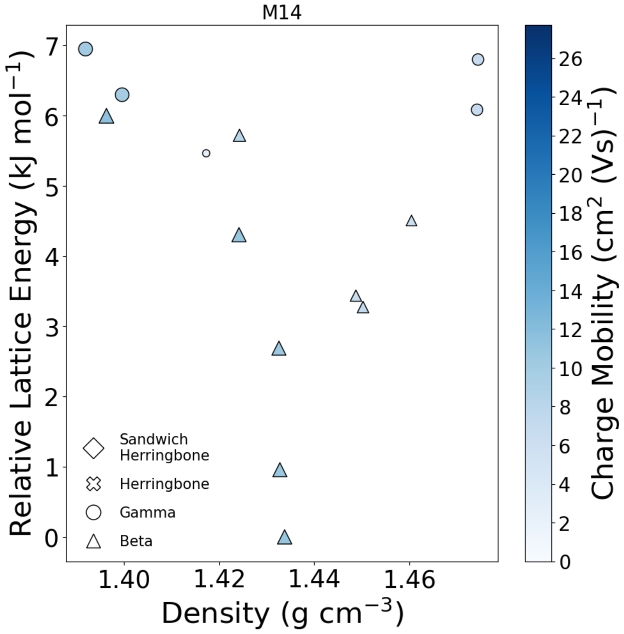

<u>Supplementary Figure 31</u> – Predicted 7.2 kJ mol^{-1} low energy window energy-density-function map for charge mobilities of each crystal structure for **M14**. Each data point represents a sampled crystal structure, with the darker and larger the data point the greater the calculated mobilities. Each structure is also classified by its packing motif.

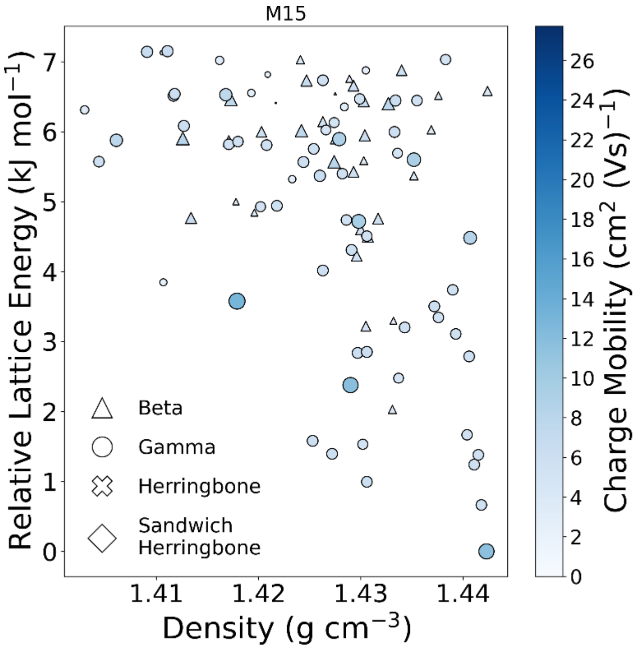

<u>Supplementary Figure 32</u> – Predicted 7.2 kJ mol^{-1} low energy window energy-density-function map for charge mobilities of each crystal structure for **M15**. Each data point represents a sampled crystal structure, with the darker and larger the data point the greater the calculated mobilities. Each structure is also classified by its packing motif.

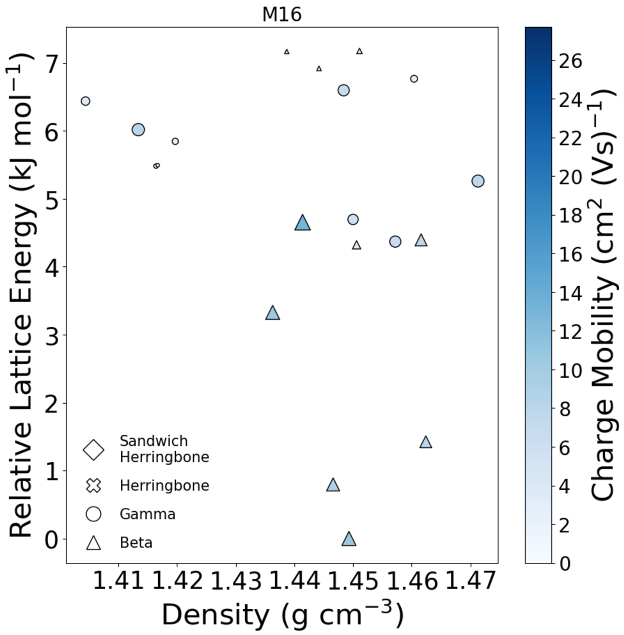

<u>Supplementary Figure 33</u> – Predicted 7.2 kJ mol^{-1} low energy window energy-density-function map for charge mobilities of each crystal structure for **M16**. Each data point represents a sampled crystal structure, with the darker and larger the data point the greater the calculated mobilities. Each structure is also classified by its packing motif.

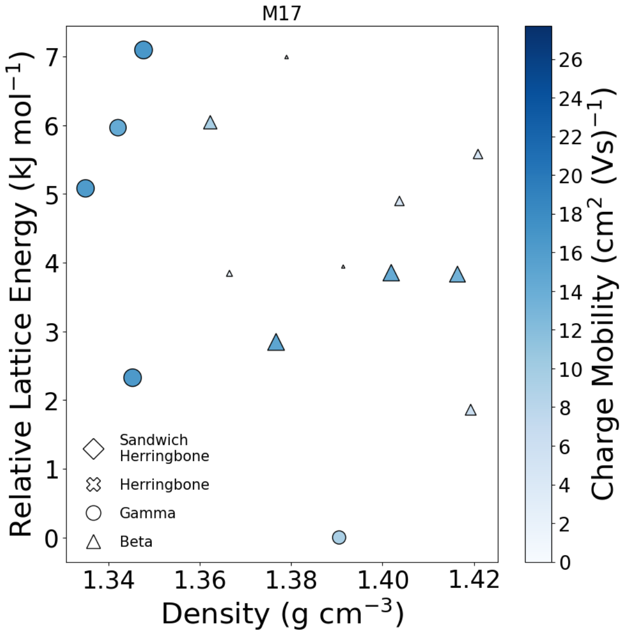

<u>Supplementary Figure 34</u> – Predicted 7.2 kJ mol^{-1} low energy window energy-density-function map for charge mobilities of each crystal structure for **M17**. Each data point represents a sampled crystal structure, with the darker and larger the data point the greater the calculated mobilities. Each structure is also classified by its packing motif.

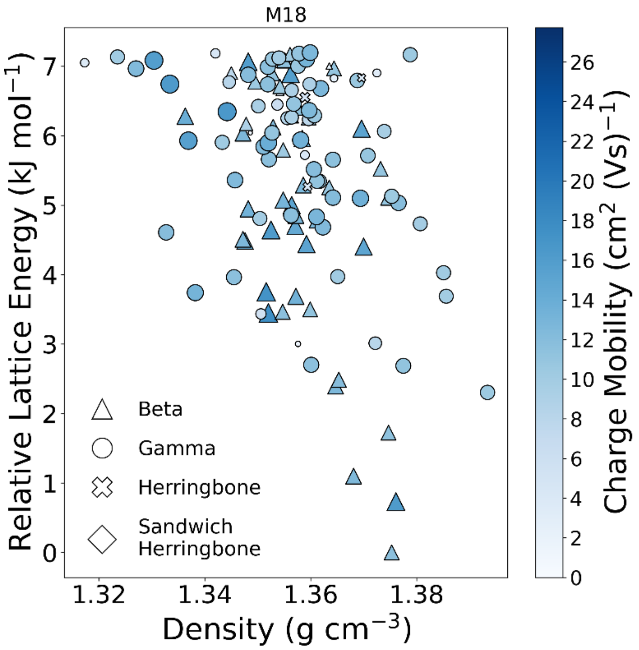

<u>Supplementary Figure 35</u> – Predicted 7.2 kJ mol^{-1} low energy window energy-density-function map for charge mobilities of each crystal structure for **M18**. Each data point represents a sampled crystal structure, with the darker and larger the data point the greater the calculated mobilities. Each structure is also classified by its packing motif.

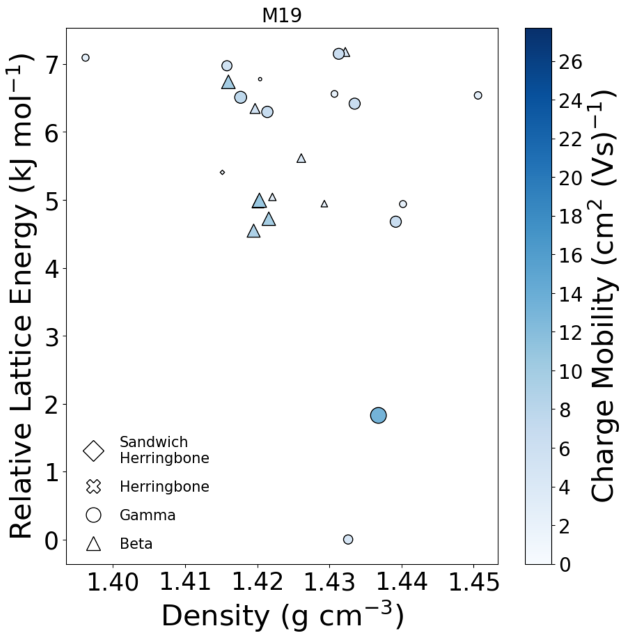

<u>Supplementary Figure 36</u> – Predicted 7.2 kJ mol^{-1} low energy window energy-density-function map for charge mobilities of each crystal structure for **M19**. Each data point represents a sampled crystal structure, with the darker and larger the data point the greater the calculated mobilities. Each structure is also classified by its packing motif.

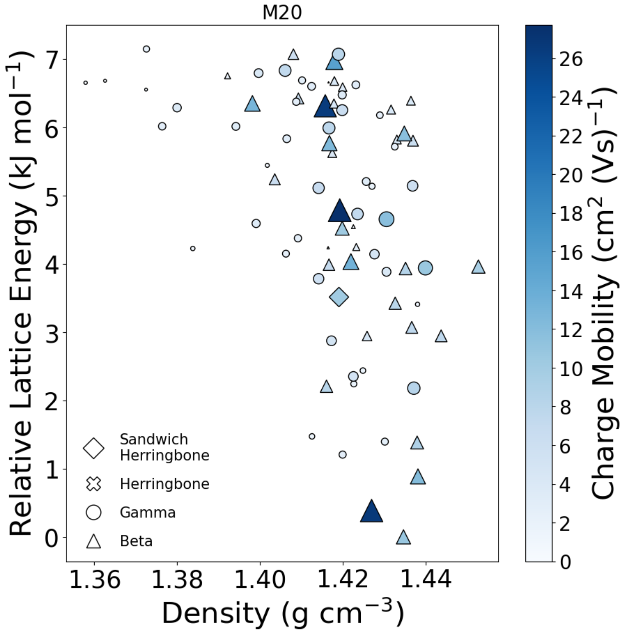

Supplementary Figure 37 – Predicted 7.2 kJ mol^{-1} low energy window energy-density-function map for charge mobilities of each crystal structure for **M20**. Each data point represents a sampled crystal structure, with the darker and larger the data point the greater the calculated mobilities. Each structure is also classified by its packing motif.

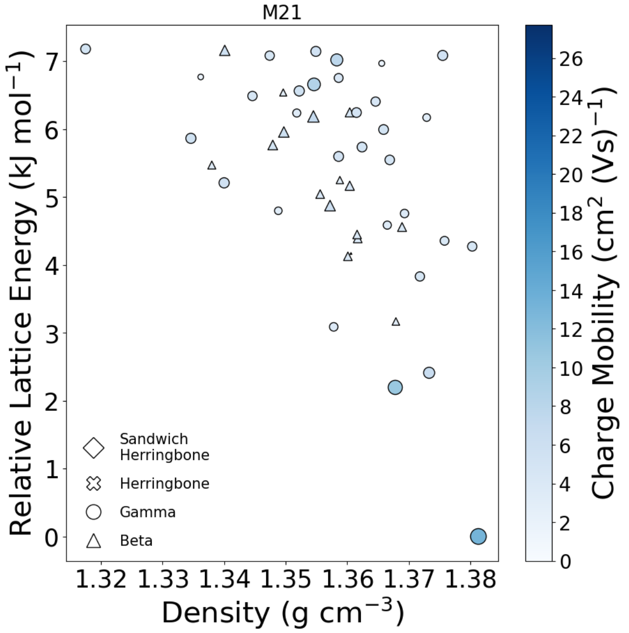

Supplementary Figure 38 – Predicted 7.2 kJ mol^{-1} low energy window energy-density-function map for charge mobilities of each crystal structure for **M21**. Each data point represents a sampled crystal structure, with the darker and larger the data point the greater the calculated mobilities. Each structure is also classified by its packing motif.

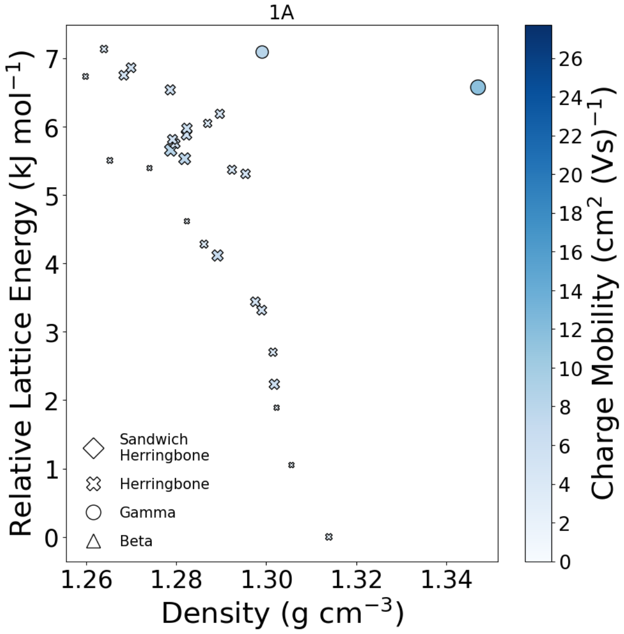

Supplementary Figure 39 – Predicted 7.2 kJ mol^{-1} low energy window energy-density-function map for charge mobilities of each crystal structure for **1A**. Each data point represents a sampled crystal structure, with the darker and larger the data point the greater the calculated mobilities. Each structure is also classified by its packing motif.

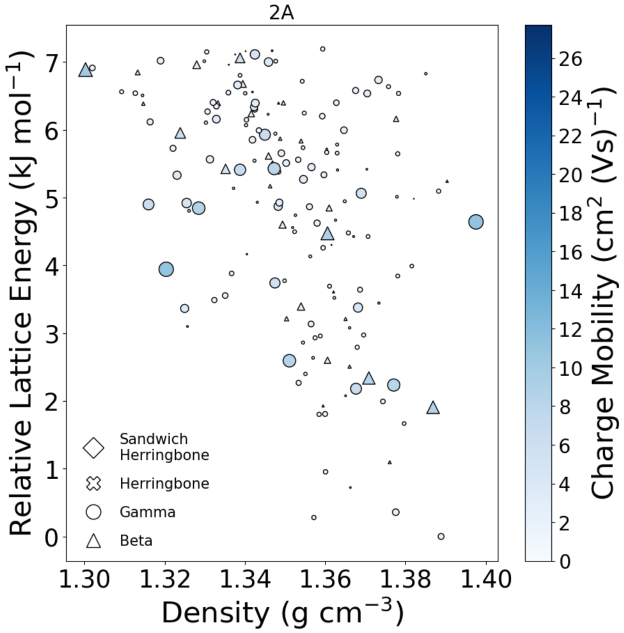

Supplementary Figure 40 – Predicted 7.2 kJ mol^{-1} low energy window energy-density-function map for charge mobilities of each crystal structure for **2A**. Each data point represents a sampled crystal structure, with the darker and larger the data point the greater the calculated mobilities. Each structure is also classified by its packing motif.

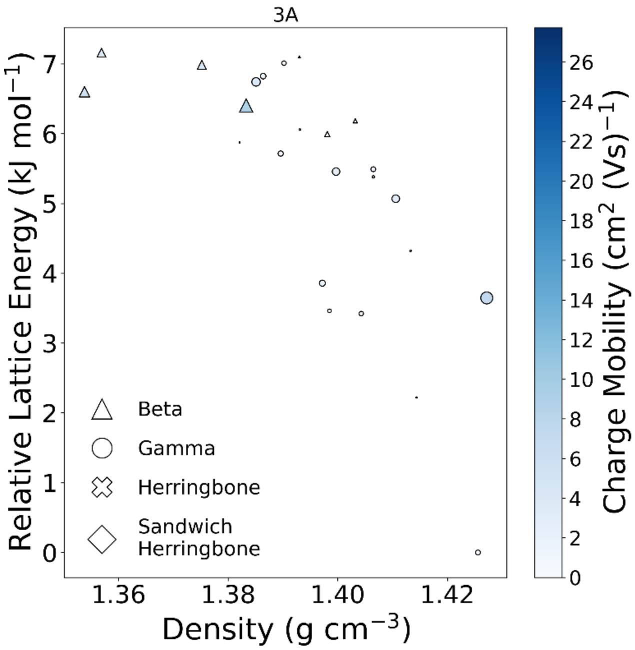

<u>Supplementary Figure 41</u> – Predicted 7.2 kJ mol^{-1} low energy window energy-density-function map for charge mobilities of each crystal structure for **3A**. Each data point represents a sampled crystal structure, with the darker and larger the data point the greater the calculated mobilities. Each structure is also classified by its packing motif.

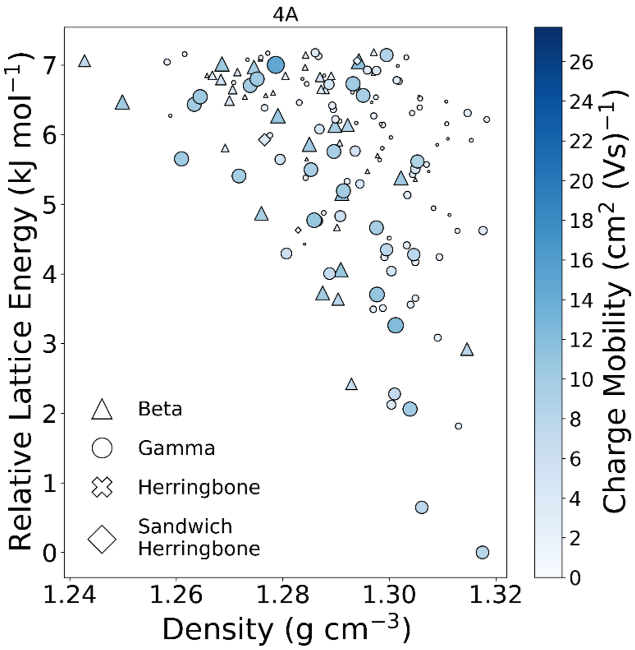

<u>Supplementary Figure 42</u> – Predicted 7.2 kJ mol^{-1} low energy window energy-density-function map for charge mobilities of each crystal structure for **4A**. Each data point represents a sampled crystal structure, with the darker and larger the data point the greater the calculated mobilities. Each structure is also classified by its packing motif.

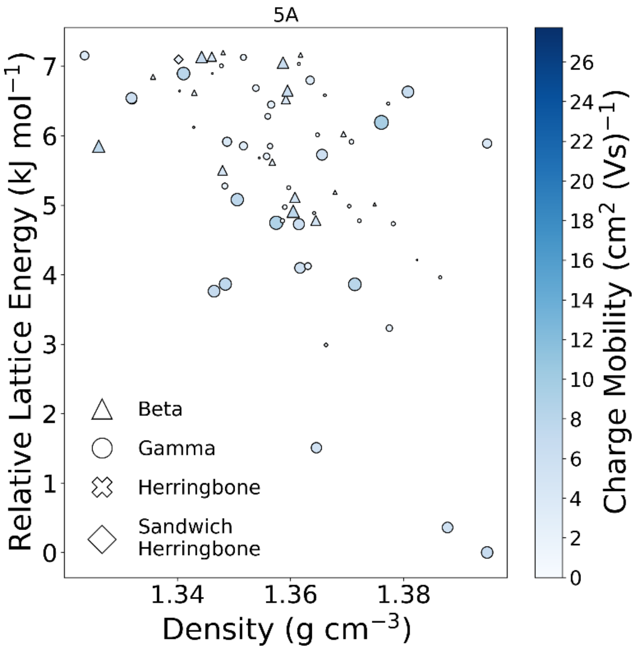

Supplementary Figure 43 – Predicted 7.2 kJ mol-1 low energy window energy-density-function map for charge mobilities of each crystal structure for **5A**. Each data point represents a sampled crystal structure, with the darker and larger the data point the greater the calculated mobilities. Each structure is also classified by its packing motif.

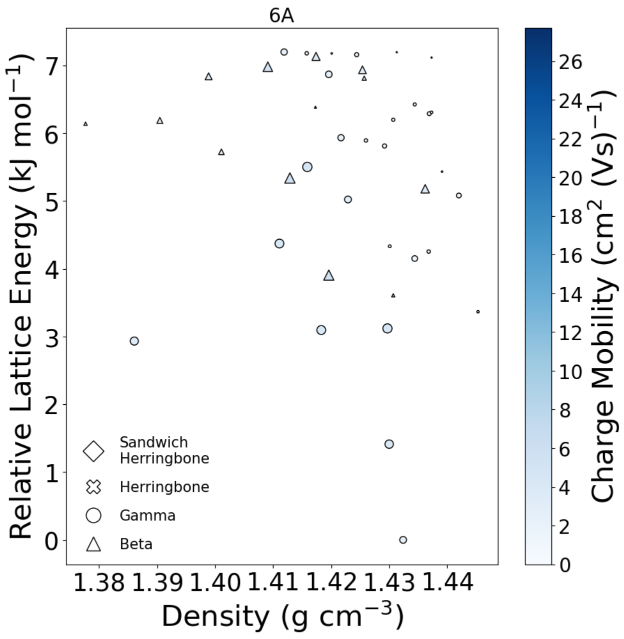

Supplementary Figure 44 – Predicted 7.2 kJ mol-1 low energy window energy-density-function map for charge mobilities of each crystal structure for **6A**. Each data point represents a sampled crystal structure, with the darker and larger the data point the greater the calculated mobilities. Each structure is also classified by its packing motif.

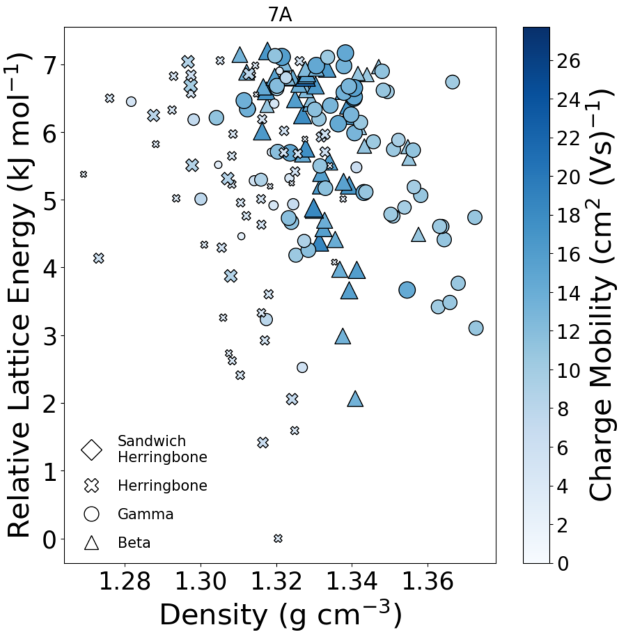

Supplementary Figure 45 – Predicted 7.2 kJ mol^{-1} low energy window energy-density-function map for charge mobilities of each crystal structure for **7A**. Each data point represents a sampled crystal structure, with the darker and larger the data point the greater the calculated mobilities. Each structure is also classified by its packing motif.

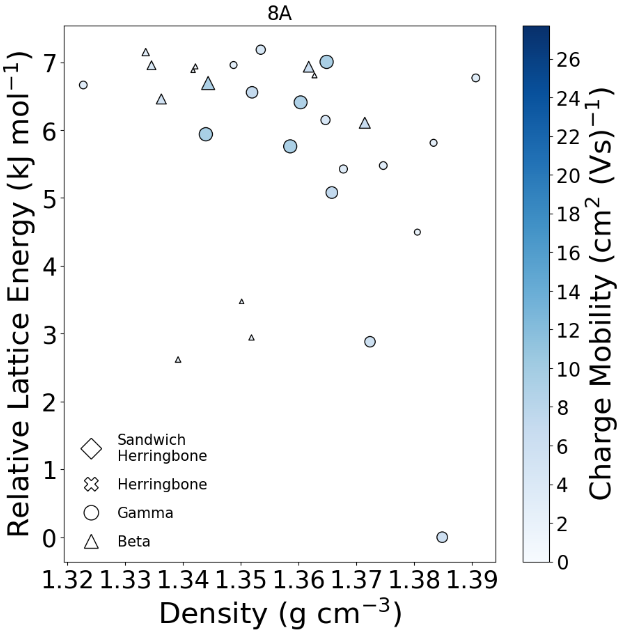

Supplementary Figure 46 – Predicted 7.2 kJ mol^{-1} low energy window energy-density-function map for charge mobilities of each crystal structure for **8A**. Each data point represents a sampled crystal structure, with the darker and larger the data point the greater the calculated mobilities. Each structure is also classified by its packing motif.

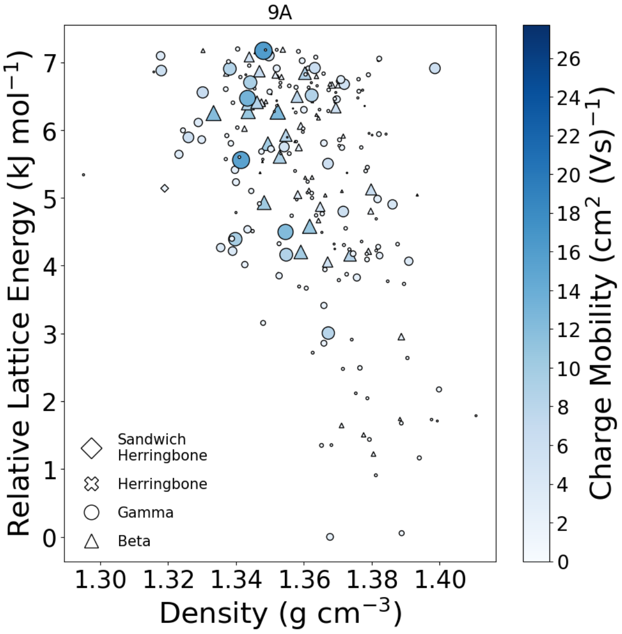

Supplementary Figure 47 – Predicted 7.2 kJ mol^{-1} low energy window energy-density-function map for charge mobilities of each crystal structure for **9A**. Each data point represents a sampled crystal structure, with the darker and larger the data point the greater the calculated mobilities. Each structure is also classified by its packing motif.

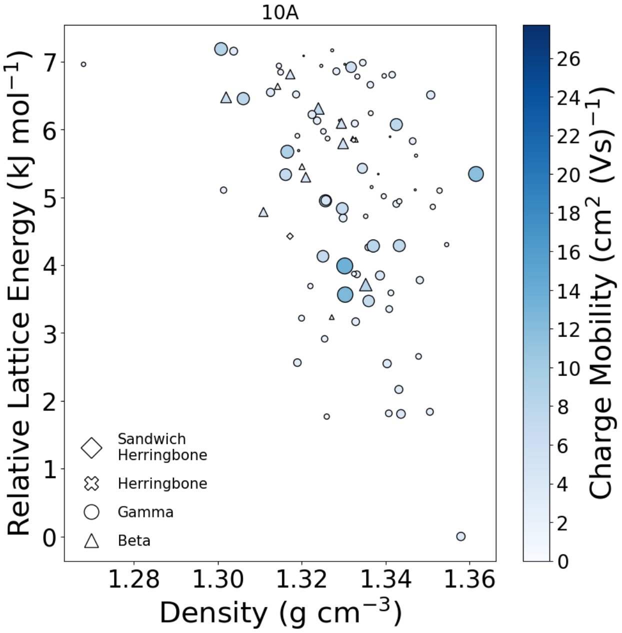

Supplementary Figure 48 – Predicted 7.2 kJ mol^{-1} low energy window energy-density-function map for charge mobilities of each crystal structure for **10A**. Each data point represents a sampled crystal structure, with the darker and larger the data point the greater the calculated mobilities. Each structure is also classified by its packing motif.

Supplementary References

1. Groom, C. R., Bruno, I. J., Lightfoot, M. P. & Ward, S. C. The Cambridge Structural Database. *Acta Crystallogr. B* **72**, 171–179 (2016).
2. Case, D. H., Campbell, J. E., Bygrave, P. J. & Day, G. M. Convergence Properties of Crystal Structure Prediction by Quasi-Random Sampling. *J. Chem. Theory Comput.* **12**, 910–924 (2016).
3. Yang, S. & Day, G. M. Exploration and Optimization in Crystal Structure Prediction: Combining Basin Hopping with Quasi-Random Sampling. *J. Chem. Theory Comput.* **17**, 1988–1999 (2021).
4. Cheng, C. Y., Campbell, J. E. & Day, G. M. Evolutionary chemical space exploration for functional materials: computational organic semiconductor discovery. *Chem. Sci.* **11**, 4922–4933 (2020).
5. Gajdos, F., Valner, S., Hoffmann, F., Spencer, J., Breuer, M., Kubas, A., Dupuis, M. & Blumberger, J. Ultrafast Estimation of Electronic Couplings for Electron Transfer between pi-Conjugated Organic Molecules. *J. Chem. Theory Comput.* **10**, 4653–4660 (2014).
6. Ziogos, O. G. & Blumberger, J. Ultrafast estimation of electronic couplings for electron transfer between pi-conjugated organic molecules. II. *J. Chem. Phys.* **155**, 244110 (2021).
7. Jiang, L., Dong, H. & Hu, W. Organic single crystal field-effect transistors: advances and perspectives. *J. Mater. Chem.* **20**, 4994–5007 (2010).
8. Jiang, H., Zhu, S., Cui, Z., Li, Z., Liang, Y., Zhu, J., Hu, P., Zhang, H.-L. & Hu, W. High-performance five-ring-fused organic semiconductors for field-effect transistors. *Chem. Soc. Rev.* **51**, 3071–3122 (2022).
9. Jiang, H., Yang, X., Cui, Z., Liu, Y., Li, H., Hu, W., Liu, Y. & Zhu, D. Phase dependence of single crystalline transistors of tetrathiafulvalene. *Appl. Phys. Lett.* **91**, 123505 (2007).
10. Moret, M. & Gavezzotti, A. The crystalline state of rubrene materials: intermolecular recognition, isomorphism, polymorphism, and periodic bond-chain analysis of morphologies. *New J. Chem.* **46**, 7626–7637 (2022).
11. Mas-Torrent, M., Hadley, P., Bromley, S. T., Ribas, X., Tarrés, J., Mas, M. Molins, E., Veciana, J & Rovira, C. Correlation between Crystal Structure and Mobility in Organic Field-Effect Transistors Based on Single Crystals of Tetrathiafulvalene Derivatives. *J. Am. Chem. Soc.* **126**, 8546–8553 (2004).
12. Nyman, J. & Day, G. M. Static and lattice vibrational energy differences between polymorphs. *CrystEngComm* **17**, 5154–5165 (2015).
13. Nyman, J., Pundyke, O. S. & Day, G. M. Accurate force fields and methods for modelling organic molecular crystals at finite temperatures. *Phys. Chem. Chem. Phys.* **18**, 15828–15837 (2016).
14. Frisch, M. J. et al. *Gaussian v.09* (Gaussian, Inc., 2009).
